# The Crude Extract from *Puerariae* Flower Exerts Antiobesity and Antifatty Liver Effects in High-Fat Diet-Induced Obese Mice

**DOI:** 10.1155/2012/272710

**Published:** 2012-05-27

**Authors:** Tomoyasu Kamiya, Mayu Sameshima-Kamiya, Rika Nagamine, Masahito Tsubata, Motoya Ikeguchi, Kinya Takagaki, Tsutomu Shimada, Masaki Aburada

**Affiliations:** ^1^Research and Development Division, Toyo Shinyaku Co. Ltd., 7-28 Yayoigaoka, Tosu-shi, Saga 841-0005, Japan; ^2^Research Institute of Pharmaceutical Sciences, Musashino University, Nishitokyo-Shi, Tokyo 202-8585, Japan

## Abstract

Kudzu, a leguminous plant, has long been used in folk medicine. In particular, its flowers are used in Japanese and Chinese folk medicine for treating hangovers. We focused on the flower of Kudzu (*Puerariae thomsonii*), and we previously reported the antiobesity effect of *Puerariae thomsonii* flower extract (PFE) in humans. In this study, we conducted an animal study to investigate the effect of PFE on visceral fat and hepatic lipid levels in mice with diet-induced obesity. In addition, we focused on gene expression profiles to investigate the antiobesity mechanism of PFE. Male C57BL/6J mice were fed a high-fat diet (HFD) or an HFD supplemented with 5% PFE for 14 days. PFE supplementation significantly reduced body weight and white adipose tissue (WAT) weight. Moreover, in the histological analysis, PFE supplementation improved fatty liver. Hepatic reverse transcription-polymerase chain reaction revealed that PFE supplementation downregulated acetyl-CoA carboxylase expression. For adipose tissue, the expressions of hormone-sensitive lipase in WAT and uncoupling protein 1 in brown adipose tissue (BAT) were significantly upregulated. These results suggest that PFE exerts antiobesity and antifatty liver effects in high-fat diet-induced obese mice through suppressing lipogenesis in the liver, stimulating lipolysis in WAT, and promoting thermogenesis in BAT.

## 1. Introduction

*Puerariae* flower extract (PFE) is a crude extract from the flowers of Kudzu (*Puerariae thomsonii*). It contains approximately 20 percent of isoflavones as the major ingredient. In Japan, PFE is utilized as nutritional supplement for treatment of hangovers and obesity.

Kudzu, a leguminous plant distributed in Japan, China, and other areas, has long been used in folk medicine. In particular, *Puerariae *flowers are used in Japanese and Chinese folk medicine for treating hangovers [1–3]. Niiho et al. confirmed that the *Puerariae lobata* flower exerts hepatoprotective effects in individuals with liver injury induced by carbon tetrachloride or a high-fat diet in animal studies [1, 3]. Recently, research of the effects of Kudzu on lipid metabolism and obesity was reported. Wang et al. confirmed that flavones derived from *Radix Puerariae* exert inhibitory effects on body weight, abdominal fat content, and lipid levels in the liver [4]. In addition, we preliminarily investigated the effect of PFE on body weight in humans and found that PFE intake might reduce body weight and abdominal fat content in mildly obese subjects [5]. However, the antiobesity mechanism of PFE is not known.

Obesity is a well-established risk factor for the development of hypertension, diabetes, dyslipidemia, and cancers, and it causes premature death [6]. An increase in visceral fat is responsible for many of the metabolic abnormalities, such as impaired glucose tolerance, insulin resistance, and increased very-low-density lipoprotein triglyceride (VLDL-TG) levels, associated with abdominal obesity [7–9]. In addition, it is reported that approximately 50% of cases of obesity involving visceral fat accumulation are complicated by fatty liver [10]. Fatty liver is a reversible condition in which triglycerides accumulate in large vacuoles in hepatocytes. Severe fatty liver is occasionally accompanied by inflammation, a situation that is referred to as steatohepatitis. When inflammation and steatohepatitis occur in people who do not drink alcohol, the condition is called nonalcoholic steatohepatitis (NASH), and it is known to correlate strongly with obesity [11]. Sources of fatty acids stored in the liver are as follows: 59% of fatty acids are stored in white adipose tissue (WAT); 26% are produced by lipogenesis in hepatocytes; and 15% are obtained from the diet [12]. Accordingly, it is considered that both reducing WAT content and suppressing lipogenesis in the liver are very important for controlling fatty liver.

WAT is a specialized connective tissue that functions as the major storage site for fat in the form of triglycerides. For use as energy, triglycerides are metabolized into fatty acids and transported by the bloodstream to tissues such as the liver and brown adipose tissue (BAT). Therefore, to prevent obesity, it is important to stimulate lipolysis in WAT and increase energy utilization in the liver and BAT.

Thus, in this study, we conducted an animal study to investigate the effect of PFE on visceral fat levels and hepatic lipid accumulation in mice with diet-induced obesity. In addition, we focused on the expression profiles of genes related to beta-oxidation and lipogenesis in the liver, lipolysis in WAT, and thermogenesis in BAT to investigate the antiobesity mechanism of PFE.

## 2. Materials and Methods

### 2.1. Experimental Materials

PFE was purchased from Ohta's Isan Co. Ltd. (Ushiku city, Japan). PFE contains 7 isoflavones (4 isoflavone glucosides: tectoridin (4.70%), tectorigenin 7-*O*-xylosylglucoside (8.37%), 6-hydroxygenistein-6,7-diglucoside (3.38%), and glycitin (0.17%); 3 aglycones: tectorigenin (0.83%), glycitein (0.10%), and genistein (0.06%)). All isoflavone standard preparations were purchased from Nagara Science Co., Ltd. (Gifu, Japan) and Tokiwa Phytochemical Co., Ltd. (Chiba, Japan).

### 2.2. Experimental Animals

All procedures using animals were performed in accordance with the Guidelines for the Care and Use of Experimental Animals of the Japanese Association for Laboratory Animal Science and were approved by the Ethical Committee of TOYO SHINYAKU Co., Ltd. Male C57BL/6J mice were purchased from Charles River Laboratories Japan Inc. (Yokohama, Japan) at the age of 6 weeks.

### 2.3. Test Environment

During the acclimation period, mice were housed in polycarbonate animal cages (260 × 420 × 180 mm; CLEA Japan, Inc., Tokyo, Japan) in groups of 4 and administered the MF diet (Oriental Yeast Co. Ltd., Tokyo, Japan). During the test period, mice were housed in individual stainless steel wire mesh cages (750 × 210 × 150 mm; Tokiwa, Tokyo, Japan) under a 12 h light : dark cycle.

### 2.4. Experimental Design

After acclimation for 1 week, C57BL/6J mice were weighed and assigned to one of 2 groups so that the mean body weight of each group was uniform. The control group was given a high-fat diet (HFD), and the treatment group was given the HFD containing 5% PFE (HFD + PFE) (Table 1). The animals were restricted-fed and given water *ad libitum* for 14 days.

### 2.5. Body Weight and Food Intake

During the experiment, the animals were weighed every 4 days. Food intake was determined daily by determining the amount of feed remaining from the previous day, and the mean daily food intake for each animal was calculated.

### 2.6. Measurement of Fecal Lipids

All feces were collected daily on days 11–14 after the start of the experiment. The collected feces were dried for at least 3 days at 100°C and weighed. After dry feces were crushed and homogenized, fecal lipids were extracted using the Folch extraction protocol [13]. The total lipid content was determined by measuring the total dry extract weight.

### 2.7. Measurement of Tissue Weight

Fourteen days after the start of the experiment, mice were sacrificed, and then the liver and interscapular brown, mesenteric, epididymal, and retroperitoneal adipose tissues were removed. All samples excluding BAT samples were weighed. Each sample was cut into small pieces, dipped in RNAlater (Ambion, Tokyo, Japan), and stored at −80°C until RNA extraction. Another piece of each liver sample was stored at −80°C for subsequent analysis.

### 2.8. Hepatic Histological Analysis

Oil Red-O staining was performed in frozen liver sections to detect the presence of fat. The degree of fatty liver was assessed by expert pathologists. We entrusted all analyses to Narabyouri Research Co., Ltd. (Nara, Japan).

### 2.9. Real-Time Quantitative Reverse Transcription-Polymerase Chain Reaction (RT-PCR)

The total RNAs from the liver, epididymal adipose tissue, and BAT were isolated using the RNeasy Mini Kit (QIAGEN, Tokyo, Japan) according to the manufacturer's directions. Total RNA (1.0 *μ*g) was reverse-transcribed into cDNA in a reaction mixture using the QuantiTect Reverse Transcription kit (QIAGEN) according to the manufacturer's directions. The gene expression levels in the liver, epididymal adipose tissue, and BAT were determined using a real-time PCR system (MiniOpticon System; Bio-Rad Laboratories, Inc., Tokyo, Japan), the QuantiTect SYBR Green PCR kit (QIAGEN), and specific sets of primers (Table 2). The relative gene expression level was calculated with real-time PCR data relative to glyceraldehyde-3-phosphate dehydrogenase (GAPDH).

### 2.10. Statistical Analysis

Data were expressed as the mean ± SEM. For comparisons between groups, analyses were performed using unpaired *t*-tests on all test items. All statistical analyses were performed using Statview ver. 5.0 (SAS Institute Japan Ltd., Tokyo, Japan), and significance was set at *P* < 0.05.

## 3. Results

### 3.1. Body Weight and Food Intake

Table 3 shows the body weight and amount of food intake 14 days after the start of the experiment. There was no significant difference between the 2 groups regarding food intake. Conversely, both the final body weight and body weight gain in the HFD + PFE group were significantly lower than those in the HFD group.

### 3.2. Liver and Adipose Tissue Weight

Table 3 shows the liver weight and epididymal, mesenteric, and retroperitoneal adipose tissue weights on day 14 after the start of the experiment. There were no significant differences between the 2 groups regarding liver weight and mesenteric adipose tissue weight. However, epididymal and retroperitoneal adipose tissue weights in the HFD + PFE group were significantly lower than those in the HFD group. These data indicate that PFE reduces body weight by decreasing fat weight in high-fat diet-induced obese mice.

### 3.3. Fecal Lipid Levels

There was no significant difference between the 2 groups regarding fecal lipid levels (Table 3). In addition, food intake was not significantly different between the 2 groups (Table 3), and thus, it was believed that energy intake did not differ substantially between the 2 groups.

### 3.4. Hepatic Histological Analysis

**Figure 1:**
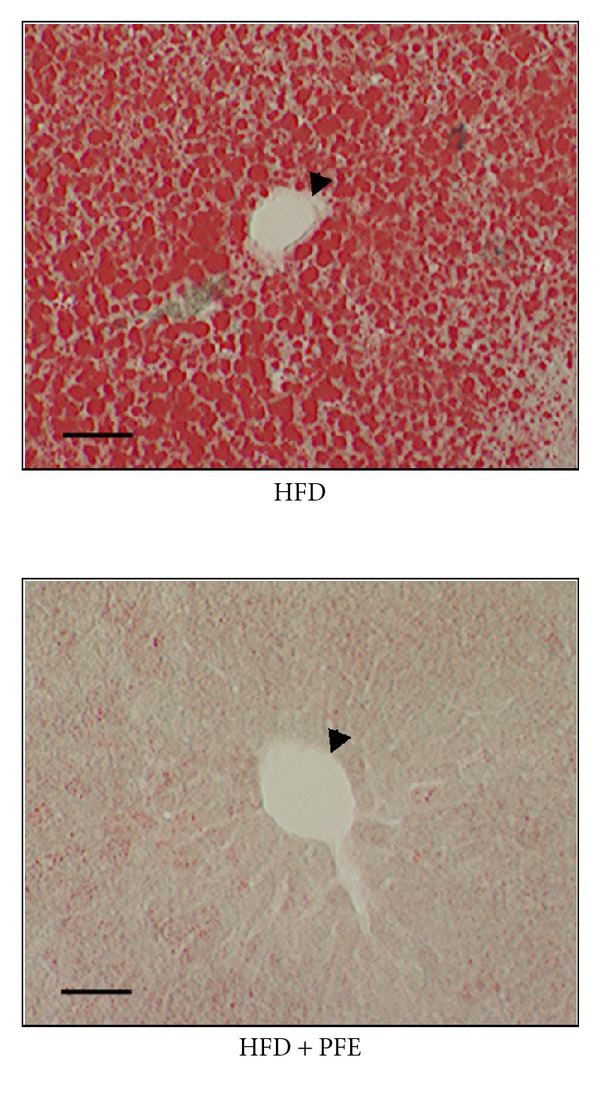
Histological analysis of the liver (Oil Red-O staining). The liver was extracted 14 days after commencing the treatment. Oil Red-O staining was performed in frozen liver sections to detect the presence of fat. Lipid droplets are stained red with Oil Red-O. The arrowhead shows the lumen of a blood vessel. These images are representative of observations made on 8 mice per group. (Scale bar: 50 *μ*m).

Table 4 and Figure 1 show the hepatic histological analysis data on day 14 after the start of the experiment. In the HFD + PFE group, the development of fatty liver was apparently suppressed.

### 3.5. Real-Time Quantitative RT-PCR

Table 5 shows the effect of PFE on mRNA expression in the liver, WAT, and BAT. The expression of hepatic genes involved in lipogenesis such as acetyl-CoA carboxylase (ACC) in the HFD + PFE group was significantly lower than that in the HFD group. For WAT, hormone-sensitive lipase (HSL) was significantly upregulated in epididymal adipose tissue in the HFD + PFE group. Similarly, uncoupling protein1 (UCP1) in BAT was significantly upregulated in the HFD + PFE group. The expression of genes related to beta-oxidation, such as carnitine palmitoyltransferase1 (CPT1), medium-chain acyl-CoA dehydrogenase (MCAD), and acyl-CoA oxidase (ACO), was not significantly different between the 2 groups, although their expressions were higher in the HFD + PFE group. These results suggest that PFE has antiobesity effects in high-fat diet-induced obese mice through suppressing lipogenesis in the liver, stimulating lipolysis in WAT, and promoting thermogenesis in BAT.

## 4. Discussion

The Kudzu flower is a rich source of isoflavones [14], and soy isoflavones such as genistein and daidzein have been reported to exert antiobesity effects [15–17]. Recently, Kim et al. reported that daidzein supplementation prevented nonalcoholic fatty liver disease in an animal study [18], and thus, it is believed that isoflavones are promising compounds for preventing obesity and fatty liver disease.

In this study, PFE supplementation significantly reduced body weight, body weight gain, and WAT weight without affecting energy intake (i.e., food intake and fecal lipid content). It is known that obesity develops when energy intake exceeds energy expenditure. Therefore, PFE was believed to exert antiobesity effects by increasing energy expenditure.

In our unpublished data, the isoflavone fraction from *Puerariae thomsonii* stimulated body weight loss in mice. Soy isoflavone is reported to exert antiobesity effects through suppressing lipogenesis in the liver by increasing protein kinase A activity [18] and promoting lipolysis in WAT by increasing cAMP levels [19, 20]. Moreover, tectoridin, an isoflavone characteristic of PFE, has been reported to modulate the expression of beta-oxidation genes such as MCAD and ACO in mice with ethanol-induced liver steatosis [21]. In this study, PFE suppressed the expression of ACC and FAS, which are rate-limiting enzymes in fatty acid biosynthesis, in the liver. In addition, the expression of HSL, the predominant lipase effector of catecholamine-stimulated lipolysis, was also upregulated in WAT. Moreover, insignificant increases in the expression of beta-oxidation genes such as CPT1, MCAD, and ACO were observed (Table 5). These results are similar to the antiobesity effects of isoflavones [18–20]; therefore, the active ingredient of PFE may be an isoflavone.

In addition, PFE supplementation significantly upregulated UCP1 expression in BAT. UCP1 is a key factor that determines the level of thermogenesis in BAT, and peroxisome proliferator-activated receptor gamma coactivator1a (PGC1a) is known to control UCP1 expression and mitochondriogenesis. A number of studies using mice revealed that UCP1 in BAT controls body fat levels by promoting energy expenditure [22–24]. Research about the effects of isoflavones on UCP1 expression in BAT is sparse; however, it is generally known that cAMP promotes UCP1 expression in BAT [25]. Therefore, PFE may promote UCP1 expression by increasing cAMP levels in BAT as observed in WAT. These results suggest that PFE affects energy expenditure; however, we have to validate this hypothesis by measuring oxygen consumption.

NASH is associated with progressive liver disease, fibrosis, and cirrhosis. Its pathogenesis is considered to include 2 steps. The first step is the development of hepatic steatosis due to the accumulation of free fatty acids in the liver, and the second step involves additional biochemical insults, including oxidative stress, the upregulation of inflammatory mediators, and dysregulated apoptosis [26, 27]. Currently, therapeutic options for NASH are limited to medications that reduce the risk factors. Therefore, suppressing hepatic lipid accumulation, the first step of the pathogenesis of NASH, appears to be very important for preventing this hepatic disorder. In this study, PFE supplementation suppressed the development of fatty liver (Table 4). In addition, genes related to lipogenesis were significantly downregulated in the liver by PFE ingestion (Table 5). Thus, PFE might be expected to prevent NASH by suppressing hepatic lipid accumulation. In fact, GOT, GPT, and gamma-GTP expressions were significantly reduced by PFE ingestion in our preliminary clinical study [5]. This result supports the possibility that PFE supplementation provides the dual effects of preventing both obesity and hepatic disorders.

## 5. Conclusion

In the present study, the flower extract of *Puerariae thomsonii* has been demonstrated to possess antiobesity and antifatty liver by in vivo assay. In addition, the flower extract of *Puerariae thomsonii* appears to exert these effects through suppressing lipogenesis in the liver and promoting lipolysis in white adipose tissue and thermogenesis in brown adipose tissue. In future research, we must clarify the detailed mechanism of PFE and its effects on energy expenditure.

## Figures and Tables

**Table 1:** Composition of the experimental diets.

Ingredient	HFD	HFD + PFE
Casein	20.0	20.0
Alpha-potato starch	28.2	23.2
Sucrose	13.0	13.0
Corn oil	20.0	20.0
Lard	10.0	10.0
Cellulose	4.0	4.0
Mineral mixture	3.5	3.5
Vitamin mixture	1.0	1.0
dl-Methionine	0.3	0.3
PFE	0.0	5.0

Total	100.0	100.0

Mineral mixture, AIN-76; Vitamin mixture, AIN-76

(g/100 g diet).

**Table 2:** Polymerase chain reaction primer sequences.

Gene name	Function	GenBank ID	Direction	Sequences
Fatty acid synthase (FAS)			Forward	TCCTGGGAGGAATGTAAACAGC
	Lipogenesis	NM___007988.3		
			Reverse	CACAAATTCATTCACTGCAGCC

Acetyl-CoA carboxylase (ACC)			Forward	TGGATCCGCTTACAGAGACTTT
	Lipogenesis	NM_133904.2		
			Reverse	GCCGGAGCATCTCATTCG

Carnitine palmitoyltransferase l (CPT1)			Forward	CTTCCAAGGCAGAAGAGTGG
	Beta-oxidation	NM_013495.2		
			Reverse	GAACCTTGGCTGCGGTAAGAC

Medium-chain acyl dehydrogenase (MCAD)			Forward	TCGAAAGCGGCTCACAAGCAG
	Beta-oxidation	NM_007382.4		
			Reverse	CACCGCAGCTTTCCGGAATGT

Acyl-CoA oxidase (ACO)			Forward	TCTTCTTGAGACAGGGCCCAG
	Beta-oxidation	AF006688.1		
			Reverse	GTTCCGACTAGCCAGGCATG

Hormone-sensitive lipase (HSL)			Forward	CCTACTGCTGGGCTGTCAA
	Lipolysis	BC021642.1		
			Reverse	CCATCTGGCACCCTCACT

Uncoupling protein 1 (UCP1)			Forward	CTGGGCTTAACGGGTCCTC
	Thermogenesis	NM_009463.2		
			Reverse	CTGGGCTAGGTAGTGCCAGTG

PPARg coactivator alpha (PGCla)			Forward	TCGATGTGTCGCCTTCTTGC
	Mitochondriogenesis	BC066868.1		
			Reverse	ACGAGAGCGCATCCTTTGG

GAPDH			Forward	ATGACATCAAGAAGGTGGTG
	Housekeeping	XM_001478412.1		
			Reverse	CATACCAGGAAATGAGCTTG

**Table 3:** Effects of PFE on food intake, body weight, liver weight, adipose tissue weight, and fecal total lipids.

	HFD	HFD + PFE
Food intake, g/day	2.83 ± 0.04	2.79 ± 0.11
Final body weight, g	26.4 ± 0.5	24.9 ± 0.3*
Body weight gain, g	2.8 ± 0.3	1.4 ± 0.3**
Liver weight, g/100 g body weight	4.54 ± 0.08	4.73 ± 0.12
White adipose tissue weight		
Epididymal, g/100 g body weight	3.22 ± 0.18	2.49 ± 0.14**
Mesenteric, g/100 g body weight	1.31 ± 0.07	1.11 ± 0.10
Retroperitoneal, g/100 g body weight	0.36 ± 0.03	0.26 ± 0.02*
Fecal total Lipids, g/day	0.015 ± 0.001	0.017 ± 0.001

The data represent the mean ± SEM values (*n* = 8).

* and ** indicate significantly different at *P* < 0.05, *P* < 0.01, respectively.

**Table 4:** Results of hepatic histological analysis.

				HFD								HFD + PFE				
				Animal no.								Animal no.				
	1	2	3	4	5	6	7	8	1	2	3	4	5	6	7	8
Fatty liver	+++	+	++	+++	+++	+++	+	+++	−	−	−	−	−	−	−	−

−: no abnormality, ±: minor, +: slight, ++: moderate, +++: severe.

**Table 5:** Effect of PFE on mRNA levels in the liver, WAT, and BAT.

	HFD	HFD + PFE
Liver		
FAS	1.00 ± 0.28	0.58 ± 0.10
ACC	1.00 ± 0.11	0.70 ± 0.06*
CPTl	1.00 ± 0.14	1.21 ± 0.14
MCAD	1.00 ± 0.23	1.51 ± 0.26
ACO	1.00 ± 0.14	1.35 ± 0.20
Epididymal adipose tissue		
HSL	1.00 ± 0.14	1.84 ± 0.15**
Brown adipose tissue		
UCPl	1.00 ± 0.23	2.15 ± 0.38*
PGCla	1.00 ± 0.15	1.30 ± 0.08

The data represent the mean ± SEM values (*n* = 8).

* and ** indicate significantly different at *P* <0.05, *P* <0.01, respectively.
